# ‘It’s not 9 to 5 recovery’: the role of a recovery community in producing social bonds that support recovery

**DOI:** 10.1080/09687637.2021.1933911

**Published:** 2021-06-09

**Authors:** Martin Anderson, Alison M. Devlin, Lucy Pickering, Mark McCann, Daniel Wight

**Affiliations:** a MRC/CSO Social and Public Health Sciences Unit, University of Glasgow, Glasgow, UK; b College of Social Sciences, Institute of Health and Wellbeing Social Sciences University of Glasgow, Glasgow, UK

**Keywords:** Addiction, recovery, alcohol, drugs, networks, identity

## Abstract

**Aim:**

To understand how the social networks of a new recovery community can help sustain recovery, focusing on processes of social identity change, in the context of the wider UK recovery movement.

**Methods:**

A cross-sectional, mixed-methods social network analysis (SNA) of ego-network sociograms to map network transitions, using retrospective measures. Ten men were recruited from a peer-worker programme, in the South Ayrshire Alcohol and Drug Partnership (ADP), West of Scotland. Network measures were compared between two timepoints, just prior to current recovery and the present time. Measures included size and density, closeness of members, and their positive or negative influence, proportion of alcohol and other drug (AOD) using and recovery peers, and extent of separate subgroups. These were complemented with qualitative interview data.

**Findings:**

There was a significant transition in network composition, with the replacing of AOD-using peers with recovery peers and a broader transformation from relationships being framed as negative to positive. However, there was no significant transition in network structure, with AOD-using and recovery networks both consisting of strong ties and a similar density of connections between people in the networks.

**Conclusions:**

The transition in network composition between pre-recovery and the present indicates a different set of social influences, while the similarities in network structure indicate that the recovery network replaced the role of the using network in providing close bonds. This helped reduce social isolation experienced in early-recovery and provided a pathway into more structured opportunities for volunteering and employment.

## Introduction

Alcohol and other drug (AOD) use is a major global disease burden (Degenhardt et al., 2018). Recently there has been a significant policy paradigm shift towards ‘recovery’, particularly in the UK, US, and Australia (Best et al., 2017), often defined as lifestyle change characterised by sobriety, health, and citizenship (Betty Ford Institute Consensus Panel, 2007), although it can be self-defined, may not require abstinence, and may be indicated by improvements in quality of life, health, relationships, housing and employment (Neale et al., 2016). The movement has been welcomed for its strengths-based approach, community building, and efforts to tackle stigma (White, 2009) but critiqued for its emphasis on abstinence and personal responsibility, which can overlook structural marginalisation (Fomiatti et al., 2017). Advocates highlight the success of the conceptually similar mental health recovery movement (Campbell et al., 2011), which emphasises the relational nature of recovery (Price-Robertson et al., 2017).

The UK grassroots recovery movement involves community-building, peer-based support, and public events that celebrate recovery (Best & Lubman, 2012). Its most ubiquitous iteration, the ‘Recovery Café’ movement, mirrors mutual-aid fellowships values of peer-support and ‘giving back’, but also aims for greater social integration, and a more formal structure of employment, training, and volunteering (Beckwith et al., 2016). In Scotland, Alcohol and Drug Partnerships (ADPs) are responsible for commissioning recovery informed strategies at the local authority level (Cunningham, 2012). In Scotland, and the rest of the UK, the concept of recovery, first advocated by user-activists challenging the top-down treatment system, is now a largely professionalised discourse that shapes policy and practice (Duke, 2013; Scottish Government, 2018). Harm-reduction approaches, although evidence-based, have been critiqued for failing to help people build the drug-free lives aspired to in government policy (McKeganey, 2012) and the shift to a strengths-based recovery approach coincided with significant funding cuts for drug and alcohol services (McPhee & Sheridan, 2020). The development of the recovery movement during a decade of austerity risks framing recovery within an austerity logic, creating a paradox between the grassroots movement for community empowerment and the more individualised recovery processes that could relocate responsibility onto marginalised communties (Roy & Buchanan, 2016). There have been similar concerns that the grassroots mental health recovery movement was adapted to accommodate the needs of government policy (Smith-Merry & Sturdy, 2013).

The formation of new identities is an important component of recovery and can involve constructing biographical narratives to distance oneself from the AOD lifestyle (McIntosh & McKeganey, 2001). Identities can also be embodied in everyday activities, involving learning to manage bodily practices more complex than the repetitive routines of AOD use (Nettleton et al., 2011). Social identity models of recovery (SIMOR, Best et al., 2016) propose that participation in recovery groups leads to the internalisation of a recovery-oriented social identity, which is associated with self-efficacy, treatment retention, and sustained recovery (Bathish et al., 2017; Beckwith et al., 2015; Buckingham et al., 2013; Dingle et al., 2015). This is similar to how, in mental health recovery, social relationships can improve symptoms by shaping people’s identities, social roles, and sense of belonging (Veseth et al., 2017). However, those who wish to construct new identities can face barriers to reintegration due to stigma and marginalisation. Relapse may then be a consequence of inability to secure a position in mainstream community life (Buchanan, 2004). Overcoming these barriers requires ‘recovery capital’, the resources required to initiate and sustain recovery (White & Cloud, 2008), such as health, finances, and relationships. Social isolation may also increase susceptibility to problem use, because dislocation from community ties can engender aimlessness, making AOD lifestyles more attractive (Alexander, 2008). Recovery proponents propose that the movement provides solutions to these key issues of relationships, resources, and stigma (White, 2009), although this approach has also been critiqued for stigmatising AOD-user networks and identities instead of addressing deeper structural determinants such as criminalisation and poverty (Fomiatti et al., 2017). Indeed, there is a consistent association between structural inequality and problem AOD use, and drug-related mortality in Scotland is twenty-three times higher in the most deprived areas than the least (McPhee et al., 2019; Parkinson et al., 2018). Rates of abstinent recovery are low, particularly for individuals who have experienced homelessness or criminal justice, less than 5% of whom were identified as achieving abstinence in the Drug Outcome Research in Scotland (DORIS) study (McKeganey et al., 2006).

Most studies on social identity models of recovery use quantitative measurement scales, limiting their ability to describe complex social identities (Bathish et al., 2017). None have measured the patterns of ties between network members or applied a mixed-methods network-mapping approach. Social network theory proposes that individual outcomes are shaped by the opportunities and constraints afforded by their network composition and structure (Valente, 2010). Smaller networks are associated with poorer health generally (Cacioppo & Cacioppo, 2014) and AOD problems specifically (Mowbray & Scott, 2015). At AOD onset, friendship networks typically fragment into AOD-user and non-user groups (Best et al., 2007) and processes of social influence and social selection mean that individual AOD-use can be predicted by the AOD-use of close friends (Meisel & Goodie, 2015; Stout et al., 2012). After treatment, individuals who return to a network of AOD users are more likely to relapse than those with an abstinent network (Hawkins & Fraser, 1987) and there is substantial evidence that recovery peers provide support that contributes to sustained recovery (Best et al., 2015; Stone et al., 2016; van Melick et al., 2013). Network interventions can alter networks to reinforce abstinence (Litt et al., 2007; Valente, 2012) and geographical relocation can weaken exposure to substance-use networks (Linton et al., 2016). Building a network of non-using friends is crucial but can take considerable time and effort (Neale et al., 2012).

The type of social capital in a network can be theorised by measuring the network structure (Borgatti et al., 1998). Network theory can tell us about, broadly, two types of social capital, ‘bonding’ and ‘bridging’ capital (Putnam, 2000). Bonding capital indicates high levels of group cohesion, social support, and robust behavioural norms, whereas bridging capital indicates access to a greater diversity of information and opportunities, with fewer behavioural restrictions. The type of social capital can be measured either through how closely connected someone is to their network members (strong ties indicate bonding, weak indicate bridging) or through the density of ties between network members (a high or low proportion of connections indicate bonding or bridging, respectively) (Crossley et al., 2015). Abstinent recovery has been associated with changes in network structure that indicate the development of additional bridging capital (Hawkins & Fraser, 1987; Panebianco et al., 2016). This study aimed to expand the conception of recovery capital and social identity using mixed methods social network analysis to study the structures associated with new recovery networks and their meanings for the people embedded in those networks.

## Research questions

This study investigated the mechanisms by which recovery communities can support identity and behaviour change. The research questions were: (1) How do social network transitions relate to changes in identity and behaviour change? (2) How do recovery communities help people to maintain recovery? (3) How can the structure and composition of social networks be measured to understand how they influence AOD use and recovery?

## Methods

### Design

A cross-sectional, mixed-methods social network approach was used (Crossley & Edwards, 2016) involving the participatory mapping of ego network sociograms during a qualitative interview (Hogan et al., 2007). Ego network analysis measures the personal network of a specific actor (ego) and the patterns of ties between the actors in the network (alters). Ego networks are useful for understanding how individual behaviour is shaped by opportunities and constraints afforded by the patterns of their social relationships, in particular the forms of social capital and influence these relationships provide (Due et al., 1999).

The pre-registered hypotheses, research questions, and analysis plans are available on the Open Science Framework (OSF): https://osf.io/dmkjq/.

### Participants

Participants (*n* = 10) were purposively sampled from the South Ayrshire *Alcohol and Drug Partnership* (ADP) where they held volunteer or professional peer-support roles. All had progressed into these roles through involvement in *Recovery Ayr,* a non-residential recovery community. Four of the ten participants reported involvement in mutual aid fellowships (R1, R3, R8, R10) and six solely used *Recovery Ayr* as their recovery support. *Recovery Ayr* was formed in 2014, part of a wave of new recovery communities (Beckwith et al., 2016). It organises a weekly recovery café and a range of support groups and activities throughout the week, such as recovery meetings, yoga, and quiz nights, and provides training and volunteering opportunties. *Recovery Ayr* is officially linked to the ADP, indicating an enthusiastic adoption of recovery philosophy in the region, and some alignment between grassroots and policy levels. Purposive sampling of male participants was used (Etikan et al., 2016) as this was a pilot study for an evaluation of *River Garden*, a residential recovery community nearby, which currently only houses men. Since participants were selected because they had thrived in this community-focused recovery setting, they cannot be considered representative of all experiences and outcomes. The value of this design is in demonstrating what successful outcomes can look like.

### Measures

Participants were asked to write the initials of people in their social network on a template consisting of four concentric circles, placing people they felt closest to in the centre and least close at the outer edge (Pahl & Spencer, 2010). They were asked to create two network maps: one showing their current network and the other a retrospective map of when they ‘first started to consider change’. Inevitably, their reflections on their pre-recovery network were shaped by their present situation. Connections between alters were drawn, measuring the patterns of alter relationships (Crossley et al., 2015). Alters were marked with coloured pens to indicate positive or negative influence, prompted by the question ‘if you are trying to make a healthy lifestyle change would they support you or make it hard for you?’, with the aim of identifying the presence of positive or negative social capital (Borgatti et al., 1998). These procedures shaped the structure and flow of the qualitative interview, focusing questions on relational issues in a manner that was structural and temporal.

### Analyses

The sociograms were coded using VennMaker then exported into R for analysis. Basic proportions were calculated of network members nominated as positive or negative influences, and as AOD-using or recovery peers, measuring the extent to which being in a social environment with others who used AOD or were in recovery was important. ‘Network size’ was a basic count of the people in each network. ‘Density’ was a calculation of the overall proportion of connections among network members out of the maximum possible number of connections. ‘Closeness’ involved assigning a rank of 1–4 to each alter, indicated by how centrally they were placed on the sociogram, and calculating a mean closeness score for each network. Lower scores indicated lower perceived emotional closeness to people in the respondent’s network. ‘Homophily’, the extent to which AOD-using or recovery peers formed subgroups, was measured by the relative density of connections within these groups compared to their connections to other groups (Krackhardt & Stern, 1988). ‘Constraint’ measured the ability of the participant to engage in otherwise unconnected social groups, while ‘transitivity’ measured how connected people were within these separate groups. ‘Betweenness centralisation’ measured the extent to which the participant occupied a central bridging position between groups. Analyses were conducted on the egonets produced by participants then these measures converted into individual-level attributes (Crossley et al., 2015) and used as dependent variables. Mean values and proportions were compared between the AOD-using (T1) and recovery (T2) networks, using a paired samples t-test for means and two proportions z-test for proportions (Walters et al., 2021). The analysis of network maps and interviews were integrated using qualitative structural analysis, a procedure where the researcher notes qualitative observations about the ways network maps are structured then conducts a thematic analysis of these together with a thematic analysis of the interview transcripts (Herz et al., 2015).

### Ethics

The study received ethical approval from The University of Glasgow, College of Medical, Veterinary & Life Sciences Ethics Committee (200170088) and was conducted in 2018.

## Results

### Sample characteristics

Participants were all men with an age range of 31–53, a median age of 41 years, and had been in recovery 2–18 years, a median of 4.5 years. Seven were in recovery from illegal drugs, such as opioids, benzodiazepines, and stimulants, two from AOD, and one from alcohol alone. All were active members of the recovery community and were employed or volunteering as peer workers.

### Social influences

Social networks in recovery were characterised by significantly improved relationships. From T1 to T2, the proportion of positive alters rose from 34.8% to 91% (X^2^ (1, *N* = 169) = 80.909, *p* < .001) and the proportion of negative alters fell from 56.5% to 4% (X^2^ (1, *N* = 169) = 56.635, *p* < .001) (Table 1).

**Table 1.** Comparison of past and current networks (proportions and means).

	T1	T2	Test statistic value	Degrees of freedom	*p* value
Proportion of alters indicated as a positive influence	34.8%	91%	X^2^ = 80.909	1	<.001
Proportion of alters indicated as a negative influence	56.5%	4%	X^2^ = 56.635	1	<.001
Proportion of alters indicated as AOD-using peers	42%	0%	X^2^ = 47.821	1	<.001
Proportion of alters indicated as recovery peers	4.3%	40%	X^2^ = 25.51	1	<.001
Mean network size (SD)	6.9 (2.92)	10 (4.67)	*t* = −1.849	9	.097
Mean Betweenness centralisation (SD)	0.097 (0.176)	0.214 (0.139)	*t* = −1.588	9	.147
Mean closeness of ego-alter connections (SD)	3.42 (0.761)	3.69 (0.317)	*t* = −1.369	9	.204
Mean constraint (SD)	0.499 (0.275)	0.360 (0.132)	*t* = 1.359	9	.207
Mean density (SD)	0.639 (0.360)	0.534 (0.248)	*t* = 0.726	9	.487
Mean homophily of AOD-using and recovery subgroups (SD)	–0.341 (0.575)	–0.252 (0.394)	*t* = −0.613	9	.555
Mean transitivity (SD)	0.763 (0.343)	0.707 (0.278)	*t* = 0.414	9	.689

**Figure 1.**
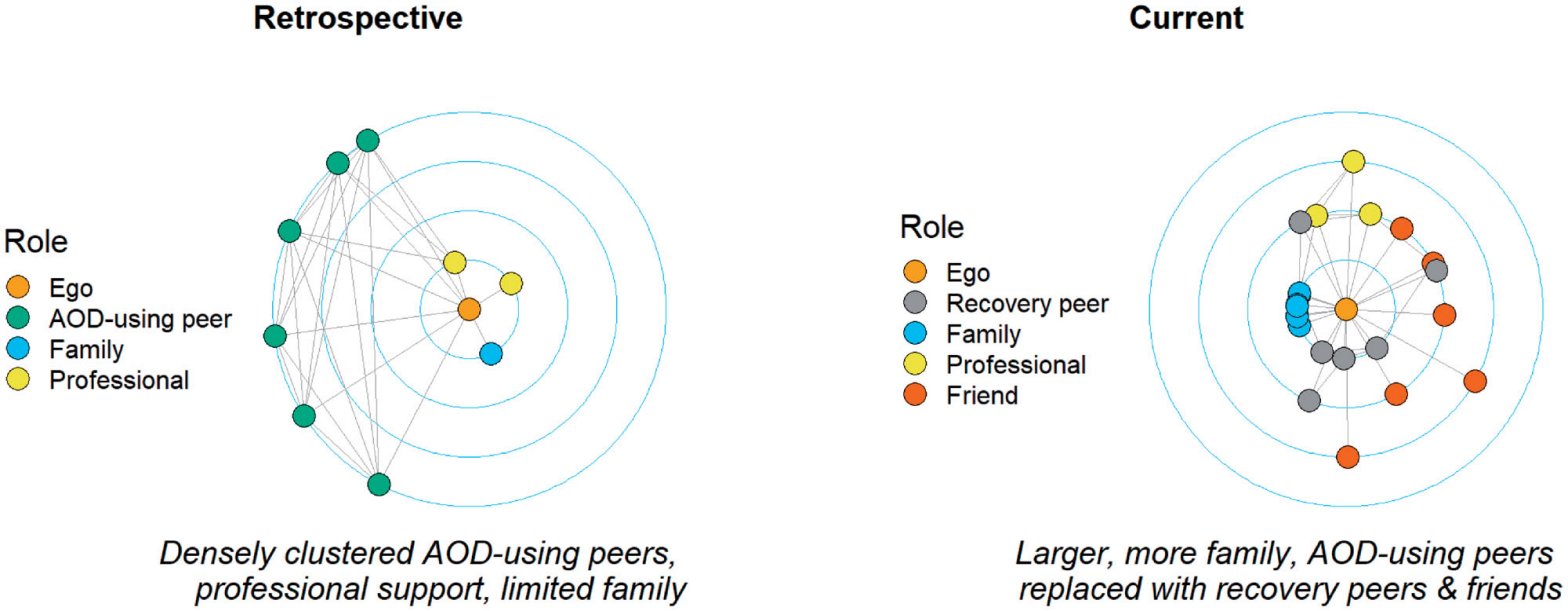
R1: comparison of retrospective and current networks.

The proportion of AOD-using peers fell from 42% at T1 to 0% at T2 (X^2^ (1, *N* = 169) = 47.821, *p* < .001) and the proportion of recovery peers rose from 4.3% at T1 to 40% at T2 (X^2^ (1, *N* = 169) = 25.51, *p* < .001). Network transition involved dropping of AOD-using peers and adding recovery peers to the network (Figure 1).

The process of transition was difficult and involved active avoidance efforts such as relocating and deleting phone numbers, sometimes producing feelings of guilt.

R2: To me, they were good pals, they were, but they weren’t supportive of my recovery … I knew I had to cut them out and I did, without telling them any reasons why I cut them out, I had to, or my recovery wouldn’t have worked.

Network influences on AOD-use were powerful. There were two primary mechanisms of influence: temptation through proximity, and active attempts to encourage AOD use and sabotage recovery.

R5: If I have a bad day or a bad time or something like that, that can lead me down a bad path … but if you’re round about people that’s actively using like that you fall into that trap.

R8: Any way they could trip me, they’d trip me up.

Two participants did report some contact with problem AOD using family members and street drinking peers and learned to negotiate new boundaries.

R2: Unfortunately, I’ve got my brothers on the same kind of negative pathway but … I can just minimise my contact with them and pick and choose which subjects I speak to them about, and I usually meet them in my mother’s house.

Participants contrasted these negative influences with their current supportive relationships. The latter included peers who were abstinent, were understanding or supportive and modelled positive behaviours, provided connections to a wider recovery network, or who were close enough to provide honest guidance and challenge negative behaviours.

R3: Even my girlfriend is in recovery because I don’t want a girlfriend that wants to … drink, I don’t want a girlfriend that wants a joint to go to sleep … I just need… positives in my life.

R5: I still struggle with depression… but I’ve got a supportive network. Instead of me going to my local dealer’s door I go to my minister’s or… somebody in recovery.

**Figure 2.**
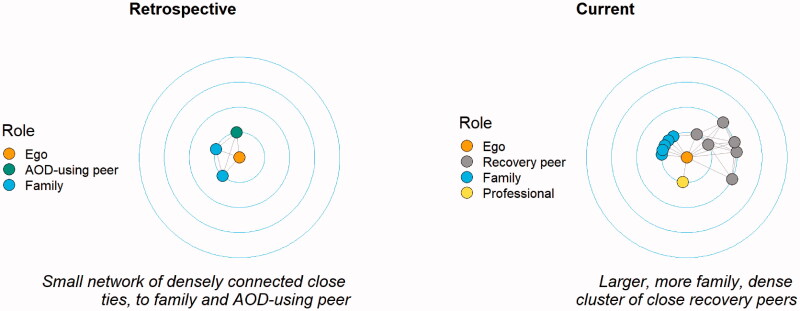
R7: comparison of retrospective and current networks.

There were no significant differences in how the networks were structured between the two timepoints, in terms of how close the participants were to their peers and how connected their peers were to each other, despite the transformation in quality of relationships and characteristics of network members. AOD-using and recovery networks both tended to involve dense connections of close ties, as in Figure 2. Both AOD-using and recovery groups formed somewhat separate subgroups, as indicated by the similarly limited connections between these groups and the wider social network (Table 1). The following quotes demonstrate the parallels between AOD-using and recovery networks.

*R4:* I stayed in a homeless hostel … And it was all the same crowd I ran about with … We were, like, co-depending on each other … they were all great pals. And they would have done anything, but all they did was … they were in the same mind as I was, want to drink and all that constantly.

R4: I’ve got two families in my life, right. So, I’ve got a family that I’ve got, like, my brother, my sister and my partner. But I’ve also got what I call my recovery family.

### Family relationships

Family relationships which were absent or negative at T1 had often become present and positive at T2. During AOD use these relationships were often characterised by limited or no contact, conflict, or dependency.

R4: Didn’t talk to any of them [family], ‘cause I couldn’t be bothered with them, ‘cause they wouldn’t give me money, so they weren’t important in my life.

Family relationships in recovery were more positive and reciprocal. Often recovery involved reconnecting with children. Some participants had been estranged from parents and reconnected during recovery, whilst others had been dependent on parents for support and found a greater sense of independence in recovery.

R1: The role of the relationships in my recovery today are really important … especially my family. Back then they were away down on my list of priorities … it was just about what I could get off [from] them. So, in terms of these relationships today they’re night and day.

The desire to rebuild relations with families was what motivated some participants to pursue recovery. For some, it was the desire to repay the support from their parents by doing better in life. Others were motivated by the need to be a good parent to their own children, particularly in cases where other primary caregivers were unable to be responsible.

R1: Even though at the time these relationships with my family were really bad I knew–I still knew–that they just wanted to see me getting better. And I think they were definitely a motivating factor.

R7: My kids … like, their mum is an addict, and they don't see their mum at all, and she’s still using. So, obviously, I wanted to come off my methadone, and I wanted to go back to school, and get myself a good job. So they’d be proud of me.

### Professional intervention and peer support

Participants reported past involvement with addiction services, psychologists, social workers, pharmacists, residential rehabilitation, hospitals, homelessness services, and prisons. One participant found prison positive because of the enforced abstinence and access to peer-support services. Four participants reported some positive experiences such as harm-reduction providing stability and good personal connections with some workers.

R8: Addiction services were important to me at that point, because they … I still had the addiction issues and that gave me the stability. See, without that, I would have needed cash … So, they were my stabiliser.

Eight participants had some negative views towards services, particularly towards impersonal relationships and overly medicalised solutions. Services were sometimes viewed as a source of drugs and a connection to fellow AOD-users.

R2: If you get that feeling that they’re only there to tick boxes and get to five o’clock in the day, you’ll lose connection with them instantly, you’re not going to get help.

R6: Everybody said, well, go and see a psychiatrist. I didn’t need a psychiatrist, I was just sad, I didn’t need medication, I went to the doctor after my gran passed away because I wasn’t sleeping, and he gave me sleeping tablets … that was like starting another addiction again.

### Social isolation

The mean network size increased from 6.9 (SD = 2.92) at T1 to 10.0 (SD = 4.67) at T2 (t(9) = −1.849, *p* = .097). Some participants had very small networks at T1 which grew at T2 due to the addition of recovery peers (Figure 2), although there were participants who had larger networks at T1 than T2. Participants with small T1 networks described social isolation and loneliness, lack of self-confidence and social skills, and a lifestyle so focused on AOD use there was no space for friendships.

R10: The last few years of my alcoholism and my drug abuse, I had no network. I didn’t want anybody near me. Absolutely nobody.

Six participants described severe isolation in early recovery after removing AOD-using peers, three of whom had not experienced isolation during AOD use. This was self-imposed to avoid anybody who might prompt a relapse.

R9: There were periods of time when I wouldn’t leave the house for four/five months at a time except to go and get the shopping … I was worried about venturing out into the big bad world again ‘cause the only people I knew were drug circles … I didn’t know how to socialise outside of those drugs circles.

### Recovery community

Belonging to *Recovery Ayr* had a central role in participants’ lives and was often contrasted to earlier, unsuccessful attempts to find support. Several mechanisms were described. First, a sense of belonging to a movement provided purpose and meaning.

R9: I got understanding, a sense of belonging and sense of it’s not just me … it might be my battle, but I’m not going through the battle alone, there’s folk having their battles and they’re all pulling in the same direction.

Second, it was a source of peer support, a democratic structure that delivered support through equal relationships. The individual became integrated into a support network not just limited to meetings and appointments.

R10: It’s not 9:00 to 5:00 recovery. Majority of the recovery time is beyond that, at night-time, with folk all sitting in the house and they can’t sleep at night and things like that … If anybody’s struggling, they lift the phone or they come in.

Third, there were opportunities for activities which provided a structure to participants’ lives and also facilitated therapeutic relationships through informal peer-support.

R4: To… be able to pick up a phone and say, look I’m struggling, or do you want go up the allotment and dig all that allotment for a couple of hours and take your frustration out on that? And talk to somebody when you’re doing it. That’s what it’s all about, rather than having that intense thing.

Finally, these activities led to opportunities for responsibility and advancement. All participants had begun attending a recovery café and progressed into peer worker roles. Having a purposeful social role, centred on using lived experience to help others, provided a foundation for recovery.

R4: I started going along to the café more and more. And then I started volunteering. And that, kind of, kept me on the straight and narrow … Started volunteering and eventually the peer worker programme came up and I thought, this is my chance … I could help folk.

The combination of a sense of belonging, peer support, activities, and opportunities for personal and professional development led to a sense of identity which radically differed from how they saw their past AOD-using selves.

R9: I used to have a nickname and that’s made it handy ‘cause the bad guy was him. And I don’t use the nickname and I haven’t used it for 20 years now … So, it’s like a different person did all that.

## Discussion

This small-scale study used mixed methods SNA to examine how, in one recovery community, identity and behaviour change were related to transitions in social networks. There were significant transitions in reported relationship quality and the proportion of AOD-using or recovery peers, some evidence that networks become larger, and striking similarities in the way networks are structured. Recovery was often motivated by a desire to rebuild relationships and required separation from AOD-using peers, enduring early-recovery isolation, then gaining new recovery peers. The study provided insights into what successful recovery can look like by retrospectively exploring the experiences of people who had already achieved successful outcomes. Consequently, caution should be exercised in generalising to those with different experiences of recovery.

### Social networks

There was a remarkable similarity between past and present networks in terms of how closely connected participants were to their networks, the people in their networks were to each other, and the extent of connections from AOD or recovery peers to the rest of the network. This unique finding shows how the recovery community replaced the structure of the AOD-use network and the transition in composition provided bonding social capital characterised by trust, support, and robust behavioural norms, at the expense of the autonomy available from networks with greater bridging capital (Crossley et al., 2015; Putnam, 2000).

This is a particularly valuable finding, as a key way the new recovery communities aimed to set themselves apart from twelve-step fellowships was to provide a bridge to wider social circles and activities (Campbell et al., 2011), which should result in more weak ties, with a reduction in scores of, for instance, constraint and transitivity. It appears that, in practice, people who find success through *Recovery Ayr* have dense networks of close ties who are also in recovery, with a similar level of separation between these peers and their wider network as their past AOD-using peers. In an AOD-using network, this type of structure can provide support but restrict opportunities to break out of AOD use; in a recovery network, this structure provides support in maintaining abstinence by restricting opportunities for relapse.

Although analyses failed to replicate findings that recovery is associated with the development of additional bridging capital (Panebianco et al., 2016), the indications of larger recovery networks are consistent with findings that network size is indicative of social capital and health (Borgatti et al., 1998; Cacioppo & Cacioppo, 2014). Therefore, it may be that recovery more generally involves bridging into wider social circles, but *Recovery Ayr* offers more resources by increasing the size of the network while operating through mechanisms of bonding capital. Indeed, participants did describe vastly expanded opportunities for activity through the *Recovery Ayr* framework of meetings and groups.

While structures were similar, when comparing AOD-using networks and recovery networks at these specific timepoints, there were significant differences in the quality of relationships and proportion of peers who used AOD or were in recovery, consistent with existing research on how networks influence AOD use (Meisel & Goodie, 2015; Stout et al., 2012) and recovery (Best et al., 2012; Bond et al., 2003). AOD-using network members were described as a powerful influence on AOD use, supporting the necessity of breaking ties (Dingle et al., 2015). Despite structural similarities, the composition of networks and the forms of social influence they provided underwent a radical transformation. This research adds to the literature by finding a wider transition in relationship quality across family, friendships, and professional relationships, mirroring the relational processes in mental health recovery (Price-Robertson et al., 2017; Veseth et al., 2017) and indicating greater social capital in the recovery networks (Borgatti et al., 1998). However, the extent to which past relationships were indicated as negative may indicate a stigmatisation of the using network that has been a critique of identity-focused recovery models (Fomiatti et al., 2017) and there were qualitative accounts that breaking ties could be an emotionally challenging process involving a substantial loss of close bonds, an aspect underemphasised in the social identity and recovery literature (Best et al., 2016; Dingle et al., 2015). It is likely that the retrospective denotation of previous network members as negative influences is an active process of ‘distancing’ (McIntosh & McKeganey, 2001) oneself from that identity rather than a true reflection of how those relations were perceived at the time.

### Early-recovery isolation

Crucially, self-imposed protective isolation in early recovery was identified as a major issue, affecting even participants who did not experience isolation during their AOD use. Even those with large AOD use networks experienced isolation when they separated from these peers in early recovery. Participants described shutting themselves away to avoid people and situations that might trigger a relapse. Research has linked AOD use to social isolation (Alexander, 2008; Beckwith et al., 2015) and the need for a non-using network (Neale et al., 2012) but this study found that social isolation was most severe in early-recovery and provides insight into the extremity and length of the isolation. This transitional period between two networks is likely to be critical for risk of relapse. The need to avoid relapse triggers is well-documented (Larimer et al., 1999) but this unsupported, do-it-yourself avoidance led to extreme social isolation and poor quality of life.

Some participants had very small, but others large, networks during AOD-use. These patterns are consistent with the theory that AOD use can offer social group membership and an associated gain in social identity for some, but a loss of relationships and social identities for others (Dingle et al., 2015), and that the subculture can offer psychosocial integration for those who are dislocated from wider society (Alexander, 2008). Serious isolation in early recovery was experienced regardless of the prior patterning of using networks.

### The recovery community

Three interrelated mechanisms by which recovery communities sustain change were identified: (1) recovery peers were closely integrated, with support delivered through peer relationships; (2) a sense of belonging and a positive recovery-oriented identity; (3) opportunities for social and professional development. These mechanisms mirror those identified in therapeutic communities: learning positive behaviours from peers with lived-experience while gaining increasing responsibilities within the community (Devlin & Wight, 2021; EMCDDA, 2014) and developing a recovery-focused social identity (Bathish et al., 2017; Beckwith et al., 2015; Best et al., 2016; Buckingham et al., 2013).

Professionals in conventional services were criticised for being impersonal and inaccessible, only allowing brief appointments during working hours. The absence of out-of-hours care was identified as a key cause of relapse. Participants admitted using services as a source of drugs but found that services were reluctant to reduce prescriptions even when they felt ready to pursue recovery. This highlights some disadvantages to individualised harm-reduction services delivered in clinical settings (Buchanan, 2004; Duke, 2013; McKeganey, 2012).

Recovery peers could provide a sense of belonging and understanding, emotional and practical support, and connections to new activities to replace the AOD-use lifestyle. This supports research finding that having recovery peers and developing a recovery-oriented social identity can predict successful recovery (Bathish et al., 2017; Beckwith et al., 2015; Best et al., 2016; Buckingham et al., 2013; Dingle et al., 2015; Frings et al., 2016). This was not explicitly an identity of ‘recovering addict’ (Frings et al., 2016) but a set of values around responsibility, community, honesty, healthy activity, and abstinence. Results support the SIMOR (Best et al., 2016) conception of social identity change linked to group membership, through which people internalise new norms and values, although evidence suggests people were motivated to change before finding the recovery group rather than being influenced through exposure to recovery peers.

Participants tended to emphasise relationships and activities rather than personal identity. This supports theories that developing new habits, practices, and routines are important in developing new ways in which identities can be embodied (Nettleton et al., 2011), and that shared meaningful activities help build the strengths and skills necessary for recovery (Johansen et al., 2013). Participants emphasised the practical opportunities they had to progress into volunteering and employment through the frameworks offered by *Recovery Ayr.*

The importance of motivation (Rollnick & Miller, 1995) was demonstrated by participants maintaining abstinence in early recovery, although motivation appeared to flow from life events rather than interventions. The recovery community provided resources for already-motivated individuals to reintegrate into community life. These social connections were important in improving quality of life (Neale et al., 2016) and sustaining long-term recovery, indicating that avoiding relapse involves securing a position in mainstream society (Buchanan, 2004).

Problem AOD use often took place in a context of homelessness, unemployment, and an overall lack of engagement in mainstream society. This highlights the importance of social exclusion, dislocation, and lack of meaningful activity (Alexander, 2008; Buchanan, 2004; Nettleton et al., 2011), which recovery communities might offer some practical resources to help overcome. These findings challenge conceptions of a disease or chronically-relapsing disorder (Kalema et al., 2019; Koob & Volkow, 2010) and indicate that addressing the social context driving AOD-use is more helpful for some people (Alexander, 2008; Best & Laudet, 2010; Buchanan, 2004; Cloud & Granfield, 2008; White & Cloud, 2008).

### Strengths and limitations

#### Strengths

The methodological approach produced useful insights into how personal networks change during recovery. The value of qualitative methods in identifying unknown phenomena and evaluating interventions (Neale et al., 2013) was borne out with insights into early-recovery isolation and some recovery community mechanisms. A unique finding was how the structure of the recovery network mirrors that of using networks but alters the composition. The negative influences were not limited to AOD-using peers and the positive influences not limited to recovery peers, demonstrating that a more comprehensive alteration of network is involved in recovery.

#### Limitations

Purposive sampling means the findings are only applicable to those with positive outcomes from engagement in the recovery community, and previous research suggests that abstinent outcomes are unlikely to be typical. Others who found recovery communities unhelpful, recovered using other types of support, or relapsed in early recovery, were not included in the sample. The sample was all-male so cannot detect gender difference. Participants were all part of the peer-worker programme and are not representative of the entire recovery community.

The sample was very small for quantitative research, reducing the statistical power to identify change. The use of retrospective measures for the AOD network was likely to have introduced recall bias, such as participants over-attributing past problems to peers or professionals. Furthermore, there was significant diversity in the length of time participants had been in recovery, and therefore how far back they had to recall their past network. Finally, the focus on relational context limited the possibility of insights into the role of structural inequality in recovery. Nevertheless, we believe that, despite these limitations, the study provides a useful insight into the social network change processes among those for whom the recovery community does work.

### Public health implications

Peer-support delivered in community settings helps some people overcome social isolation and exclusion. Harm-reduction remains vital, but it is essential that interventions offering new ways of life are available for when life events motivate change. Recovery communities are a valuable community resource which could be integrated into specialist provision (Mericle, 2014). Network interventions can help people alter their networks in ways that are conductive to recovery (Hunter et al., 2017; Litt et al., 2007; Valente, 2012). It may be valuable to target network interventions towards people in early-recovery when they are likely to be most isolated.

## Conclusions

The structures of the recovery networks appeared to mirror those of the using networks, but the transformation in network composition transformed the behavioural influence of bonding capital. The change in composition was not simply confined to adding recovery and removing AOD-using peers but involved a more radical transformation of relationship quality over multiple domains. Social isolation was particularly severe in early recovery. The recovery community first offered a solution to this isolation then sustained longer-term recovery by providing a structure of opportunities for volunteering and employment. Longitudinal research would reduce recall bias, and prospective sampling of a more representative sample of people attempting recovery would capture more variation in experiences. More research on the isolation of early recovery and how new networks form could help develop interventions. It would be useful for future research to go beyond describing changes in network composition to analysing the processes by which this happens.
